# Cognitive dysfunction in patients with interstitial lung disease

**DOI:** 10.3389/fmed.2026.1739386

**Published:** 2026-03-25

**Authors:** Xiaojun Deng, Xingquan Xiong, Yuyan Liu, Shuying Jia, Fang Wang, Xiao Guo, Shuyi Gu

**Affiliations:** 1 Department of Critical Care Medicine, Shanghai Sixth People’s Hospital, Shanghai, China; 2 Innovation and Incubation Center (IIC), Shanghai Pulmonary Hospital, School of Medicine, Tongji University, Shanghai, China; 3 Department of Pulmonary and Critical Care Medicine, Ruijin Hospital, Shanghai Jiao Tong University School of Medicine, Shanghai, China; 4 Rehabilitation Medicine College, Shandong Second Medical University, Weifang, China; 5 Department of Respiratory and Critical Care Medicine, Institute of Respiratory Medicine, Shanghai Pulmonary Hospital, Tongji University School of Medicine, Shanghai, China

**Keywords:** cognitive dysfunction, interstitial lung disease, lung-brain axis, Mini-Mental State Examination, RNA sequencing

## Abstract

**Objective:**

To investigate cognitive status in patients with interstitial lung disease (ILD) and its association with lung tissue transcriptomic alterations, and to propose potential lung-brain interaction mechanisms and clinical implications.

**Methods:**

We enrolled 45 ILD patients and 45 age-matched controls and compared Mini-Mental State Examination (MMSE) total and subscale scores. Baseline laboratory and pulmonary function characteristics of ILD were summarized. Using lung tissue RNA-seq data from GSE213001 {29 ILD cases [20 idiopathic pulmonary fibrosis (IPF), 9 non-IPF], 14 non-diseased controls [NDC], totaling 139 samples}, we performed PCA, differential expression analysis using the limma-voom framework with the duplicate Correlation function to account for within-donor correlations (threshold |log_2_FC| > 1, FDR-adjusted *p*-value < 0.05), and GO enrichment. We focused on enrichment related to neurodegeneration/cognition and inflammatory pathways, and compared the expression of AD-related genes (PSEN1, PSEN2, BACE1, and MME) between ILD and NDC.

**Results:**

ILD patients showed significantly lower MMSE total scores than healthy controls, with notable declines in attention/calculation and orientation. At the transcriptomic level, PCA clearly separated ILD from NDC, whereas IPF and non-IPF did not form distinct subgroups. Differential analysis identified 1,544 DEGs (1,142 upregulated; 402 downregulated). Enrichment analysis confirmed strong signals for inflammatory and fibrotic pathways. In an exploratory analysis, we also observed enrichment for terms related to nervous system function. The expression trends of several genes previously implicated in neurocognitive contexts, including PSEN1, PSEN2, BACE1, showed a directional concordance with patterns described in neurodegenerative contexts.

**Conclusion:**

This study provides preliminary evidence linking ILD to cognitive impairment on screening and identifies intriguing overlaps between lung tissue transcriptomic alterations and pathways relevant to brain function. These convergent observations lend biological plausibility to, and motivate further investigation of, a lung-brain axis hypothesis in ILD. The findings highlight the need to consider cognitive health in ILD management and warrant validation in longitudinal cohorts with detailed neuropsychological phenotyping.

## 1 Introduction

ILD encompasses a heterogeneous group of diffuse parenchymal lung disorders characterized by varying degrees of inflammation and fibrosis that progressively impair gas exchange and respiratory mechanics (1). The clinical course often features insidious onset of exertional dyspnea and nonproductive cough, with many patients ultimately experiencing respiratory failure. Although inflammatory processes may dominate early phases, sustained injury commonly culminates in irreversible fibrotic remodeling. ILD encompasses the spectrum of idiopathic interstitial pneumonias (IIPs), including IPF, idiopathic nonspecific interstitial pneumonia (NSIP), acute interstitial pneumonia (AIP), idiopathic lymphoid interstitial pneumonia (LIP), and cryptogenic organizing pneumonia (COP) (2). ILD may also represent pulmonary involvement of underlying systemic diseases, such as connective tissue diseases (CTD) or sarcoidosis. With ongoing industrialization and urbanization, the burden of air pollution continues to rise, long-term exposure to high concentrations of fine particulate matter (PM2.5), sulfur dioxide, and other pollutants is closely associated with the onset and progression of ILD, potentially triggering or exacerbating disease by inducing oxidative stress, chronic airway inflammation, and remodeling of the pulmonary interstitium (3). Meanwhile, occupational inhalational exposures remain important etiologic factors, with risks showing a dose—response relationship and synergistic harm with smoking. In addition, unhealthy lifestyle behaviors are associated with increased ILD risk, potentially contributing to pathogenesis by aggravating airway inflammation, injuring the alveolar epithelium, and disrupting reparative processes. IPF is the prototypical and most prevalent fibrotic ILD, and despite advances in antifibrotic therapy, prognosis remains poor (4). Contemporary ILD diagnosis integrates high-resolution CT imaging, clinical phenotyping, and pulmonary function testing (PFT), typically adjudicated by multidisciplinary discussion (5, 6). However, because early respiratory symptoms overlap with prevalent conditions such as asthma, chronic obstructive pulmonary disease (COPD), and heart failure, diagnostic delays are common. These delays can worsen outcomes by allowing disease progression before targeted therapies and supportive interventions are implemented. ILD frequently coexists with systemic comorbidities, including gastroesophageal reflux disease, sleep-disordered breathing, cardiovascular disease, diabetes, lung cancer, pulmonary hypertension, and mood disorders (7, 8). While the systemic burden of ILD is increasingly recognized, the neurocognitive consequences remain underexplored. Evidence from related respiratory conditions, particularly COPD and pulmonary hypertension, consistently links these diseases to impaired attention, memory, executive function, and processing speed, along with increased rates of depression and anxiety (9, 10). Because cognitive impairment is associated with poorer self-management, increased disability, and higher mortality in other chronic diseases, clarifying its prevalence and determinants in ILD is clinically important.

Integrating clinical phenotypes with lung transcriptomic profiles has proven valuable for characterizing disease biology and heterogeneity across chronic respiratory conditions (11, 12). In this retrospective study, we evaluated whether patients with ILD exhibit measurable cognitive deficits relative to healthy peers. We conducted a cross-sectional, case–control analysis comparing MMSE performance between clinically characterized ILD patients and age-matched and education-matched controls. Pulmonary indices were recorded to characterize the clinical profile of the patient cohort. Group comparisons and regression analyses were performed.

Therefore, we conducted a cross-sectional, case—control analysis to compare cognitive screening performance (using the MMSE) between clinically characterized ILD patients and age and education-matched healthy controls, aiming to explore potential links with lung tissue transcriptomic alterations.

## 2 Methods

### 2.1 Study design and ethics

This observational study was conducted at the Shanghai Pulmonary Hospital from October 2009 to January 2024. The study aimed to assess and compare the cognitive function status between ILD patients and healthy controls. All participants provided written informed consent. The study was approved by the Ethics Committee of Shanghai Pulmonary Hospital (approval No. K21-316).

### 2.2 Participants

**Figure 1:**
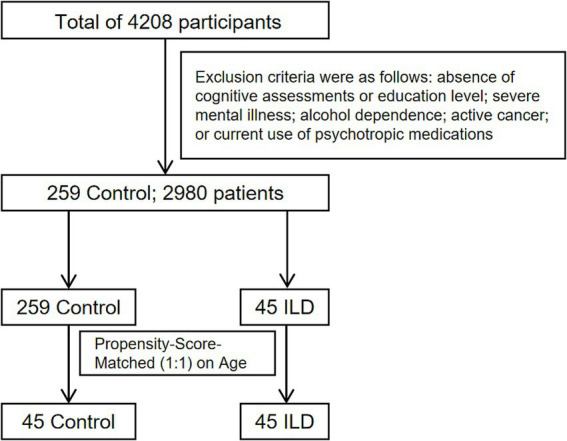
Road map for screening people.

A total of 4,208 participants were initially considered, comprising patients from Shanghai Pulmonary Hospital and individuals undergoing health examinations (who served as controls). The Participants with insufficient data (cognitive function assessments, education level record), severe mental illness, alcoholism, and those using psychotropic medications and patients with active cancer (newly diagnosed cancer or metastatic cancer) were excluded. Following this exclusion, 259 control participants and 2,980 patients remained. From the patient group, 45 individuals with a diagnosed interstitial lung disease (ILD) were identified. To minimize confounding by age, we performed propensity score matching (1:1) between these 45 ILD patients and the pool of 259 eligible controls. The propensity score was estimated using a logistic regression model with age as the sole covariate. Matching was executed via the nearest neighbor method without replacement and without a caliper. Post-matching balance was assessed and confirmed (e.g., standardized mean difference for age < 0.1). This process yielded our final analytical cohort of 45 matched control participants and 45 ILD patients (Figure 1).

The diagnosis of interstitial lung disease (ILD) was established according to current international guidelines and clinical statements (13, 14). Specifically, the diagnostic criteria for idiopathic pulmonary fibrosis (IPF) and progressive pulmonary fibrosis adhered to the official ATS/ERS/JRS/ALAT clinical practice guideline (13). All enrolled ILD patients were comprehensively diagnosed by specialists in respiratory and critical care medicine, based on a combination of clinical presentation (e.g., progressive dyspnea, dry cough), characteristic radiographic findings on high-resolution computed tomography (HRCT) (such as reticulation, honeycombing, traction bronchiectasis), and pulmonary function tests (indicating restrictive ventilatory defect and/or reduced diffusing capacity).

### 2.3 Cognitive assessment

**Figure 2:**
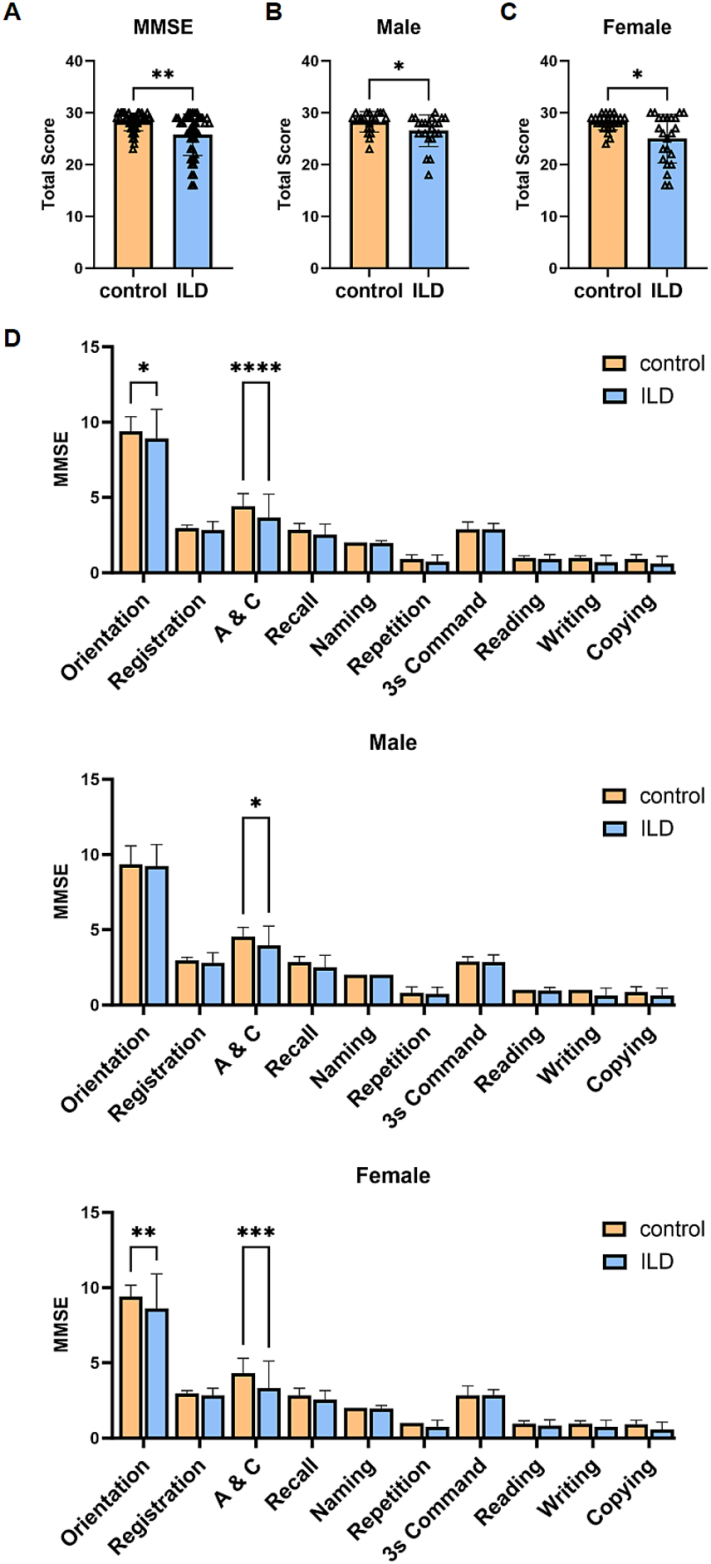
Cognitive dysfunction in patients with ILD and control subjects. **(A)** MMSE total scores in patients with ILD and control subjects. **(B)** MMSE total scores in male patients with ILD. **(C)** MMSE total scores in female patients with ILD. **(D)** The details of MMSE in patients with ILD and control subjects. Statistical analyses were conducted using Mann–Whitney U test, multiple comparisons and performed tests using the Šídák method.

Population demographic and clinical data were collected, and multiple circulating biomarkers were tested, including blood cell classification counts. Cognitive function was evaluated employing the Chinese version of the MMSE. The MMSE is a widely used global cognitive screening instrument, with total scores ranging from 0 to 30; higher scores indicate better cognitive performance. In this study, cognitive impairment was defined as an MMSE total score below 27. In addition to the total score, we conducted a detailed analysis of performance on specific cognitive sub-items. As visually summarized in Figure 2D, these sub-items were: Orientation, Registration (immediate recall), Attention and Calculation (A&C), Recall (delayed recall), Naming, and Language.

### 2.4 RNA-Seq data analysis

RNA-seq data were obtained from the Gene Expression Omnibus (GEO) database under accession number GSE213001. This dataset comprises 139 lung tissue samples derived from 43 individuals (14 non-diseased control donors and 29 patients with ILD). The pre-compiled raw count matrix was downloaded and processed in *R* (v4.5.0). To account for the non-independence of multiple samples from the same donor, differential expression analysis was performed using the limma-voom framework. Specifically, we utilized the limma package (v3.60.0) in conjunction with the voom transformation. The within-donor correlation was estimated using the duplicate Correlation function and incorporated into the linear model by specifying the donor ID as a blocking variable in the model fitting process (lmFit). The linear model included disease status (Group, ILD *vs.* Control) and technical processing batch as fixed effects. The explicit design formula was: design <−model.matrix (~ Group + batch, data = colData). The batch variable was encoded directly from the metadata field characteristics_ch1.16 (sample processing date) of the original dataset, with each unique date treated as a distinct categorical level. Genes with an absolute log2 fold change greater than 1 and a false discovery rate (FDR)-adjusted *p*-value (padj) of less than 0.05 were considered differentially expressed. Gene Ontology (GO) enrichment analysis was conducted on the resulting gene set using the cluster Profiler package (v4.16.0), with significance defined as an FDR (padj) < 0.05.

### 2.5 Statistical analysis

The Shapiro–Wilk test was used to verify data normality. Normally distributed continuous variables were summarized as mean ± SD, non-normally distributed variables as median [25th-75th percentile]. Non-parametric data were compared using the Mann—Whitney U test, while parametric data were analyzed using an unpaired *t*-test (Welch’s correction). Categorical variables were presented as counts (percentages). Comparisons were made using the Chi-square test or Fisher’s exact test (when expected frequencies were low). Participants with any missing data in the key variables of interest (MMSE scores, age, etc.) were excluded from the analysis. The cognitive function of subjects was measured by MMSE. Mann–Whitney *U* test were used to compare MMSE scores. For comparisons across multiple MMSE subscales, *p*-values were adjusted for multiple comparisons using the Sidak method. Logistic regression was used to identify factors associated with the group status (ILD *vs.* controls). Receiver operating characteristic (ROC) curve analysis was performed to evaluate the ability of the MMSE total score in discriminating between ILD patients and controls. All significance levels were *p* < 0.05. Data were analyzed with SPSS 29.0.

## 3 Results

### 3.1 Baseline characteristics

Table 1 presents the baseline characteristics of patients with ILD, including 45 patients with ILD and 45 healthy controls matched to the inclusion criteria. The two groups showed no significant differences in sex, body mass index (BMI), education level, or the comorbidity of diabetes (*p* > 0.05). However, the control group had a higher prevalence of hypertension compared to the ILD group (53.3% *vs.* 24.4%, *p* < 0.05). For the ILD group, pulmonary function measures [median (IQR) unless stated] were: FVC: 1.97 (1.16–2.60) L; FEV1/FVC: 79.72 (74.70–85.72) %; DLCO: 5.16 (3.71–7.48) mmol/min/kPa (units as per lab); DLCO/VA: 1.56 (0.99–2.37) mmol·min^−1^·kPa^−1^·L^−1^ (Table 2).

**Table 1:** Baseline characteristics in patients with ILD and control individuals.

	Control (*n* = 45)	ILD (*n* = 45)	*p* value
Sex, *n* (%)			
Male	20 (44.4%)	21 (46.7%)	0.833
Female	25 (55.6%)	24 (53.3%)	
Age at recruitment, years	68.00 (61.00–72.00)	68.00 (62.00–74.00)	0.639
BMI, kg/m^2^	23.75 ± 4.02	23.75 ± 4.03	0.346
Education*	3.00 (2.00–4.00)	3.00 (3.00–4.00)	0.576
Comorbidities^†^, *n* (%)			
Hypertension	24 (53.3%)	11 (24.4%)	0.009
Diabetes	2 (4.4%)	4 (8.9%)	0.677

BMI, body mass index; Values are means (±SD), medians (interquartile range), or *n* (%). *The degrees of education, 1 = illiteracy; 2 = primary schools; 3 = junior middle school; 4 = high school or technical secondary school; 5 = junior college; 6 = university; 7 = master’s degree or above.

†Comorbidities only include hypertension, and diabetes.

**Table 2:** Clinical characteristics of ILD.

	ILD (*n* = 45)
FVC, L	1.97 (1.16–2.60)
FEV1/FVC (%)	79.72 (74.70–85.72)
Dlco, mmol/min/kPa	5.16 (3.71–7.48)
DL_CO/_VA, mmol·min^−1^·kPa^−1^·L^−1^	1.56 (0.99–2.37)

FVC, forced vital capacity; FEV1/FVC, forced expiratory volume in 1 s/FVC; Dlco, diffusing capacity of the lung for carbon monoxide; DL_CO/_VA, DLco corrected for Alveolar Volume; Values are means (±SD), medians (interquartile range), or *n* (%).

### 3.2 Association between cognition and ILD

In terms of overall cognitive status, patients with ILD showed significantly lower total MMSE scores than healthy controls (Figure 2A), This reduction was observed regardless of sex (Figures 2B,C), indicating a general reduction in global cognitive screening scores associated with ILD. *p*-values for comparisons across the MMSE subscales were adjusted for multiple testing using the Sidak method. Analysis of MMSE subscales revealed significant declines specifically in the domains of Orientation [effect size = −0.468, 95% CI (−0.893, −0.040)], and Attention and Calculation [effect size = −0.778, 95% CI (−1.204, −0.351)]. Sex-stratified analysis of MMSE subscales revealed distinct patterns of domain-specific impairment: In male patients, significant declines were specifically observed in the domains of Orientation and Attention and Calculation [effect size = −0.596, 95% CI (−1.160, −0.031)]. In female patients, the effect sizes for the deficits in Orientation [effect size = −0.791, 95% CI (−1.423, −0.159)] and Attention and Calculation [effect size = −0.972, 95% CI (−1.604, −0.340)] were larger than those observed in male patients. While mean scores in other domains such as Repetition, Writing, and Copying were also lower in the ILD group, these differences did not reach statistical significance. These findings indicate that ILD is associated with deficits in global cognitive screening scores, with significant impairments in attention/executive function and orientation. Deficits in other domains, such as language and visuoconstruction, may also be present but require further investigation.

**Figure 3:**
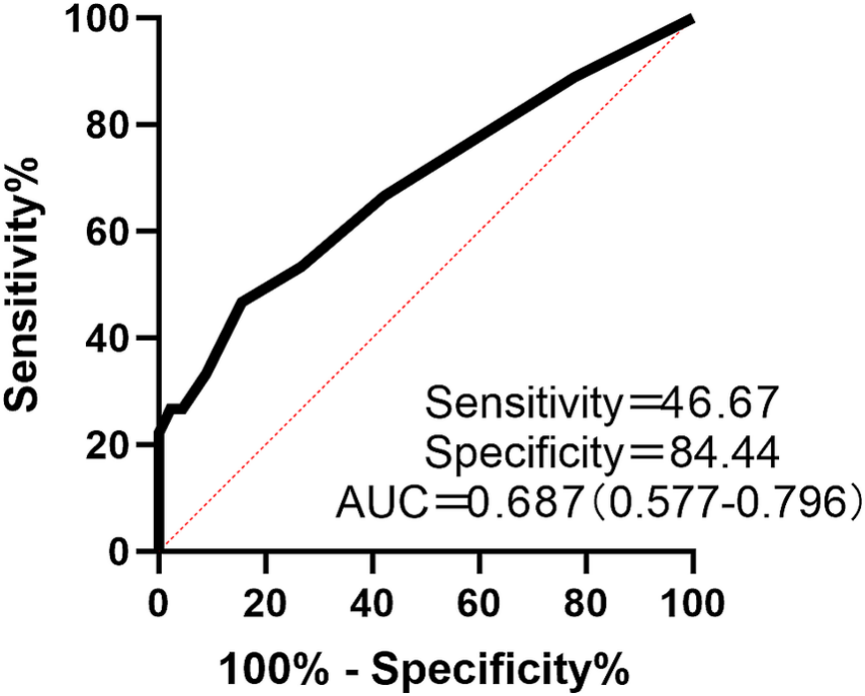
ROC curve for the MMSE total score in discriminating between ILD patients and controls.

To identify factors associated with group status (ILD vs. controls), logistic regression analyses were performed. Univariate analysis indicated that the MMSE total score, along with subscales including Attention and Calculation, Recall, Repetition, Writing, and Copying, were significantly associated with ILD status (all *p* < 0.05; Table 3). And, in the multivariate model adjusting for education, sex and hypertension, the MMSE total score was further confirmed an independent predictor (adjusted OR: 0.734, 95% CI: 0.591–0.871, *p* = 0.002). The ROC curve for the MMSE total score in discriminating between ILD and controls had an AUC of 0.687 (0.577–0.796) (Cutoff = 26.50), indicating modest discriminative ability (Figure 3).

**Table 3:** Logistic regression analysis results of 45 healthy people with control and 45 patients with ILD.

Parameters	Univariate analysis		Multivariate analysis	
	OR (95%CI)	*p* value	OR (95%CI)	*p* value
Age	1.013 (0.971–1.056)	0.553		
BMI	0.991 (0.874–1.123)	0.991		
Sex	0.836 (0.365–1.916)	0.836	0.728 (0.286–1.854)	0.507
Education	1.08 (0.765–1.526)	0.661	1.091 (0.748–1.590)	0.652
Total score	0.735 (0.606–0.891)	0.002	0.745 (0.610–0.909)	0.004
Orientation	0.808 (0.598–1.092)	0.166		
Registration	0.398 (0.1–1.593)	0.193		
A&C	0.591 (0.4–0.872)	0.008		
Recall	0.343 (0.14–0.84)	0.019		
Naming	0	0.991		
Repetition	0.268 (0.079–0.91)	0.035		
3 s Command	1 (0.401–2.494)	1.000		
Reading	0.182 (0.02–1.623)	0.127		
Writing	0.05 (0.006–0.403)	0.005		
Copying	0.188 (0.062–0.566)	0.003		
Hypertension	0.283 (0.115–0.694)	0.006	0.323 (0.123–0.847)	0.022

### 3.3 Exploratory analysis of lung transcriptomes in ILD

**Figure 4:**
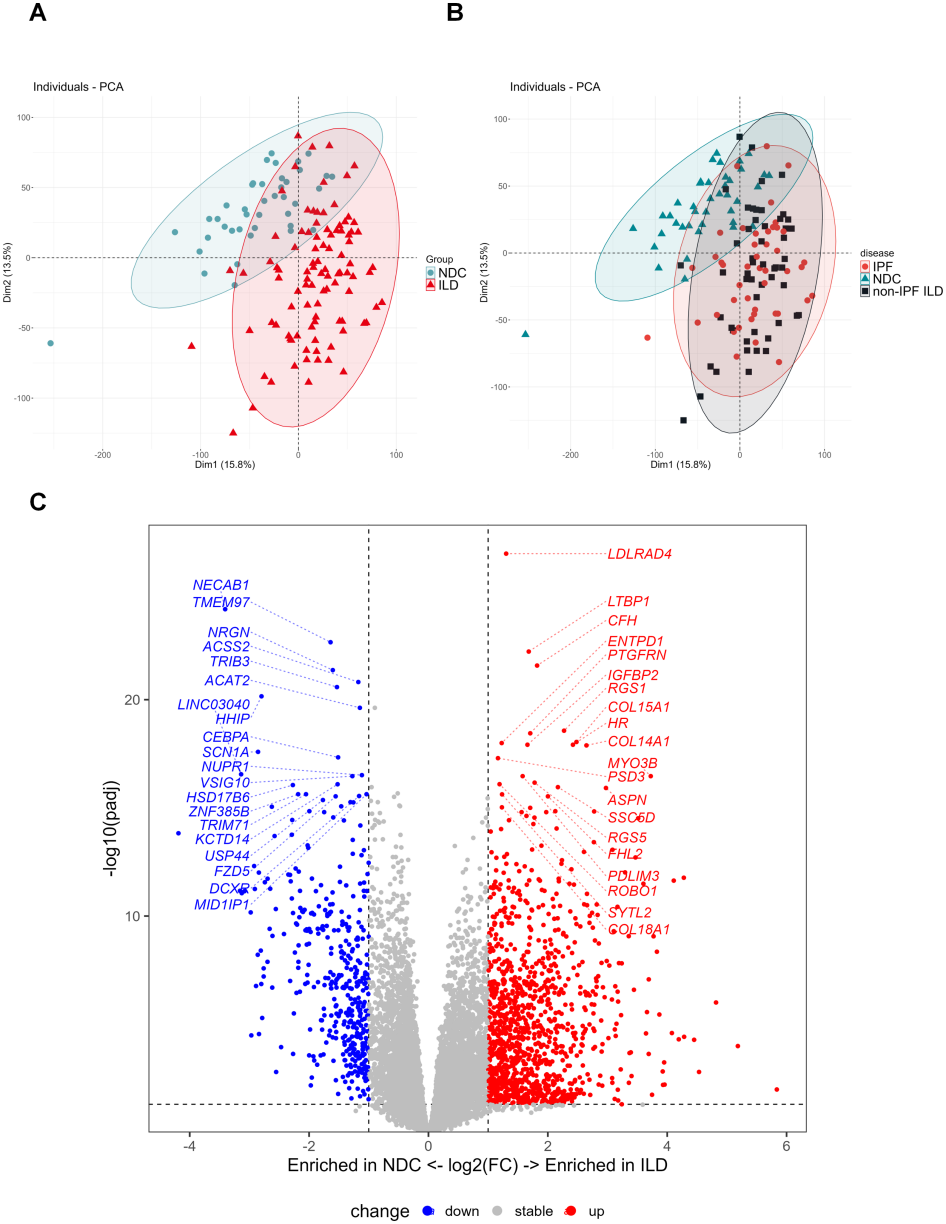
Evaluation of sample grouping and identification of differentially expressed genes. **(A,B)** Principal component analysis (PCA) plot of individual samples. **(C)** Volcano plot showing the differentially expressed genes between ILD and NDC (differentially expressed genes were defined as |Log_2_FC| > 1 and padj (FDR) < 0.05).

The RNA-seq data analyzed in this study were sourced from the publicly available dataset GSE213001, which comprises 139 lung tissue samples from 43 individuals (14 non-diseased control donors, NDC; 29 patients with ILD). To account for the non-independence of multiple samples from the same donor, differential expression analysis was performed using the limma-voom framework, with within-donor correlation estimated by the duplicate Correlation function and donor ID specified as a blocking variable. Unsupervised principal component analysis revealed clear transcriptional separation between ILD (combined IPF and non-IPF) and NDC samples, whereas IPF and non-IPF ILD samples largely overlapped (Figure 4A), suggesting shared dysregulated pathways in advanced ILD.

**Figure 5:**
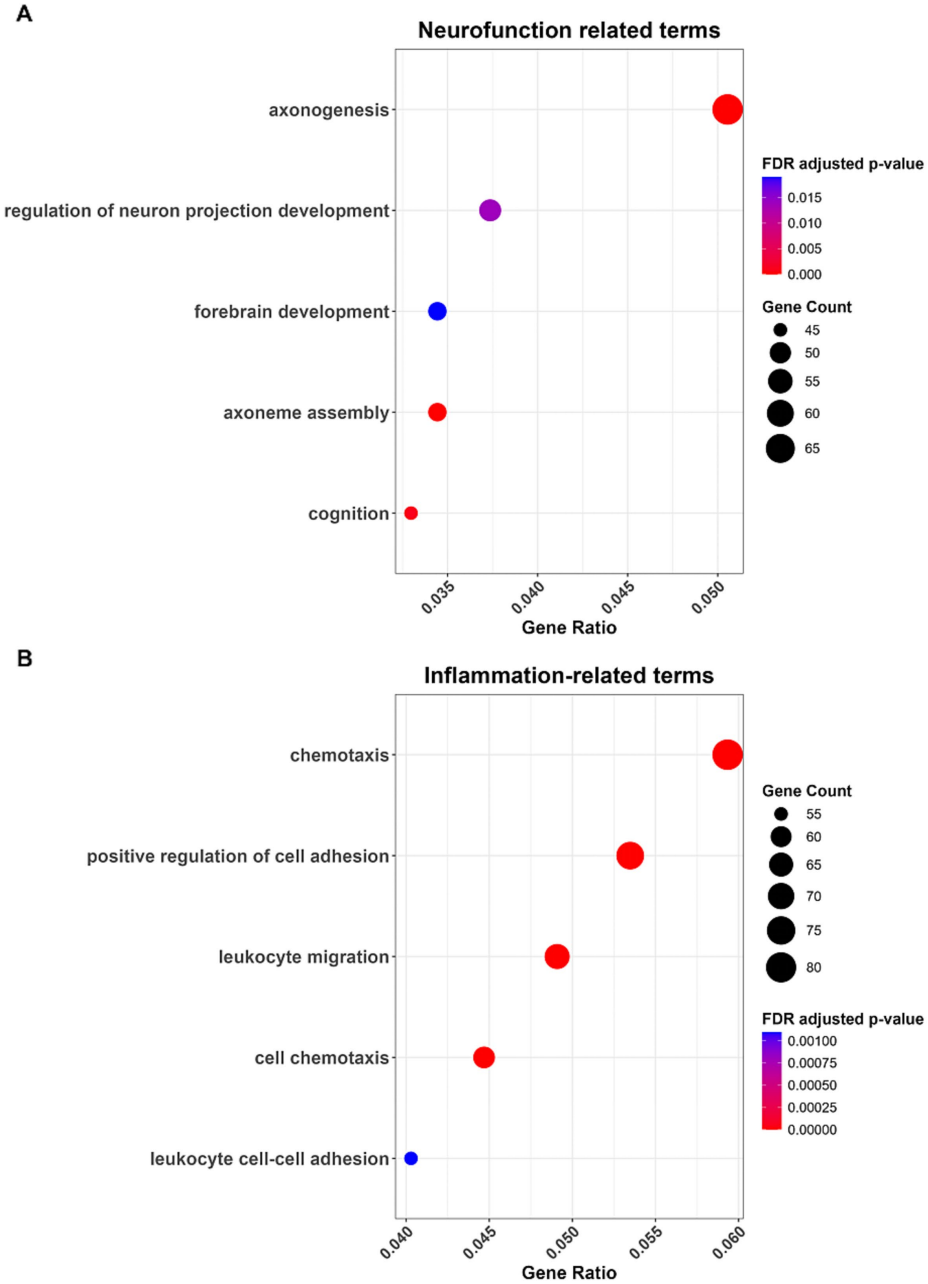
Gene ontology enrichment analysis of differentially expressed genes highlights significant enrichment in neurofunction **(A)** and inflammation-related **(B)** biological processes.

Comparing ILD to NDC samples under this corrected statistical model identified 1,544 differentially expressed genes (DEGs; 1,142 upregulated, 402 downregulated) at an FDR-adjusted *p*-value < 0.05 and |log2 fold change| > 1 (Figure 4B). Gene Ontology enrichment analysis of these DEGs revealed significant overrepresentation of pathways central to ILD pathogenesis, including “chemotaxis,” “extracellular matrix organization”, and “leukocyte migration” (Figure 5A and Supplementary material). Notably, in an exploratory analysis, we observed enrichment for biological pathways annotated with terms related to nervous system development and function (Figure 5B).

**Figure 6:**
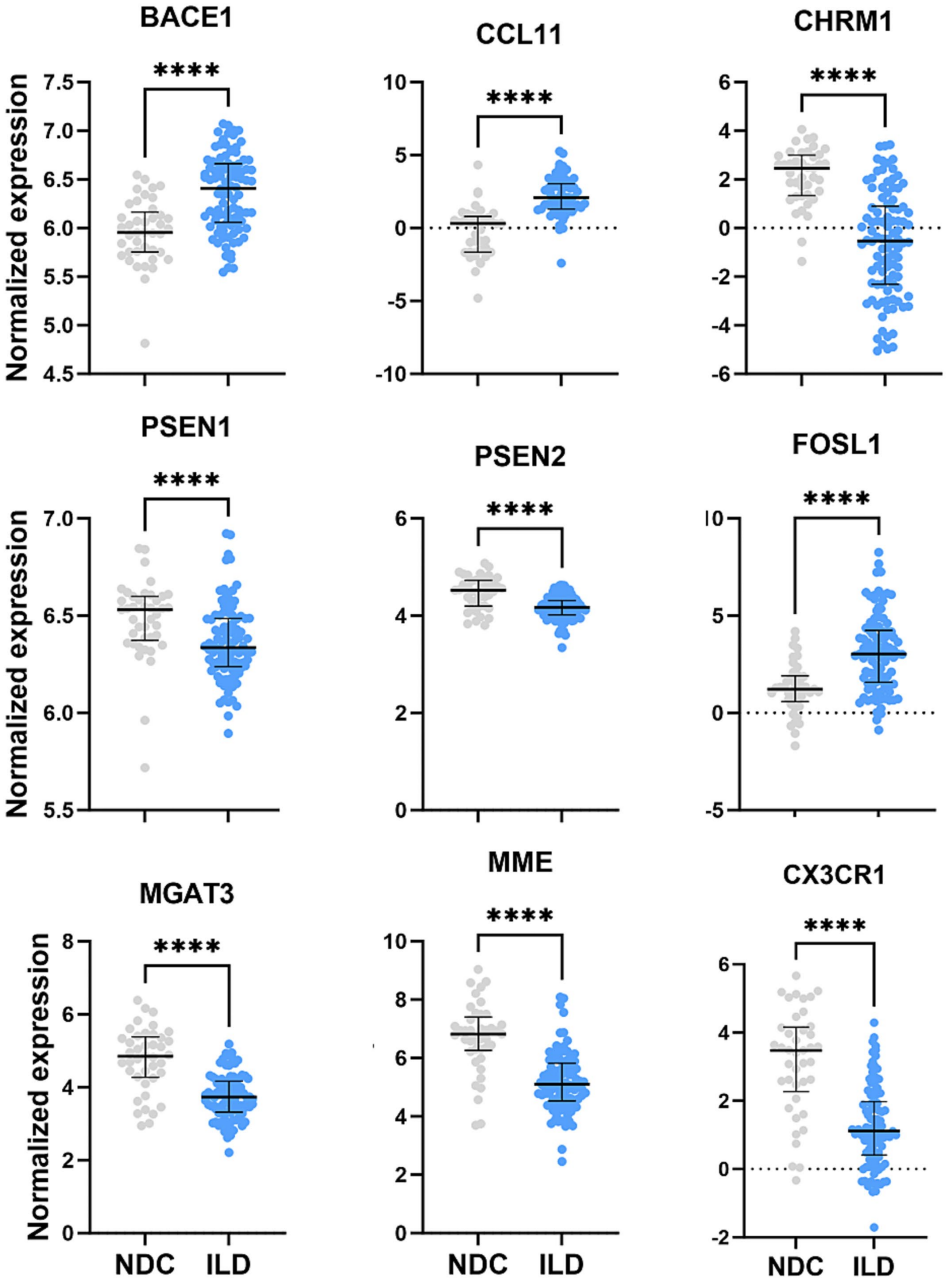
Normalized expression of genes related to coginition between ILD and NDC. Data shown are median (5th–95th percentile). Statistical analyses were conducted using Mann–Whitney *U* test.

**Figure 7:**
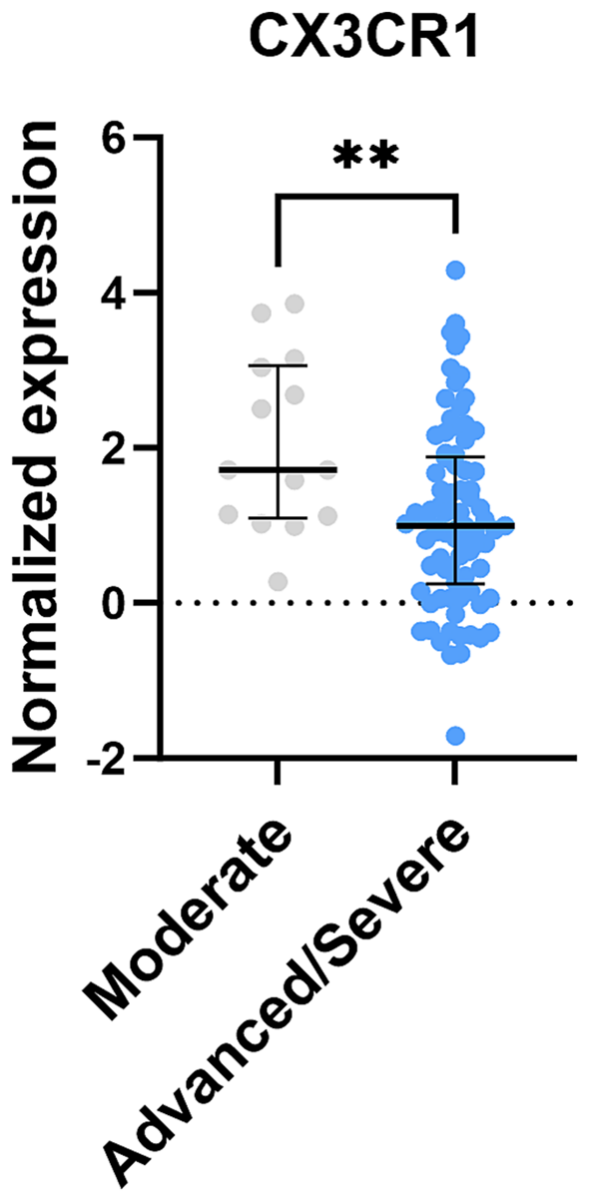
Normalized expression of genes related to coginition between moderate and advanced/severe ILD patients. Data shown are median (5th–95th percentile). Statistical analyses were conducted using Mann–Whitney *U* test.

In a hypothesis-generating follow-up, we examined the expression of a curated set of genes with prior known links to Alzheimer’s disease (AD) pathogenesis (e.g., PSEN1, PSEN2) and broader cognitive processes. The direction of expression changes for several of these genes in ILD lung tissue showed concordant trends with patterns reported in neurodegenerative contexts (15, 16)(Figure 6). While this exploratory analysis cannot establish causality or specific function in lung tissue, it provides a molecular correlate that aligns with the broader hypothesis of shared systemic disturbances in ILD. As an exploratory analysis, we also assessed whether the pulmonary expression of neurologically relevant genes correlated with clinical indicators of disease severity in the public dataset. The expression of CX3CR1 was lower in patients with advanced disease compared to those with moderate disease (Figure 7), lending preliminary biological plausibility to, and motivating further investigation of, the hypothesis of a lung-brain axis in ILD (Table 3).

## 4 Discussion

This study enrolled 45 patients with ILD and 45 healthy controls, and found that ILD patients had significantly lower overall MMSE scores, with deficits specifically concentrated in the domain of Attention and Calculation. Despite the limited sample size and the exploratory nature of the study, our findings remain informative given the current lack of systematic reports on cognition in ILD. Overall, the ILD group had lower MMSE scores than the control group. Sex-stratified analyses showed differences between male ILD patients and male controls, as well as between female ILD patients and female controls. These findings highlight cognitive impairment as a potential, yet under-investigated, extra-pulmonary manifestation of ILD. However, we emphasize that the causal direction, underlying mechanisms, and clinical significance of this association require confirmation through prospective, longitudinal studies with detailed neuropsychological assessments.

The potential mechanisms linking ILD to cognitive impairment are likely multifactorial, involving both direct and indirect pathways. First, systemic consequences of ILD—such as chronic and exertional hypoxemia, intermittent nocturnal desaturation from sleep-disordered breathing, vascular dysfunction and small-vessel cerebrovascular injury, systemic inflammation and oxidative stress, medication effects, and the indirect impacts of fatigue, anxiety, and depression-represent well-established risk factors for cerebral injury and cognitive impairment. Second, direct neuro-immune communication via the lung-brain axis provides a plausible framework for more immediate crosstalk (17, 18). In ILD, the pulmonary neuro-immune milieu can sense local inflammation and generate signals that are transmitted via vagal afferents to the brainstem, thereby modulating central regulatory circuits and neuroinflammatory status, which in turn may affect cognition and other brain functions. Conversely, impaired brain function may adversely influence lung physiology, as the lung appears particularly vulnerable following brain injury (19); thus, we speculate that coexistent cognitive impairment could accelerate ILD progression. Third, emerging evidence implicates the respiratory and gut microbiota in the regulation of systemic inflammation and neuro-modulator production (e.g., serotonin, melatonin) (20, 21). Alterations in the microbiome of ILD patients might thereby contribute to cognitive changes through the gut-lung-brain axis, which represents a novel, testable mechanism that warrants future investigation. Given shared clinical features between ILD and COPD, a similar trajectory of cognitive vulnerability in ILD is plausible. Collectively, these intertwined pathways underscore the need for integrated management strategies that address both pulmonary and cognitive health in ILD.

The clinical interpretation of our findings requires caution. The modest discriminative ability of the MMSE total score (AUC = 0.687, 95% CI: 0.577–0.796) in distinguishing ILD patients from controls underscores that the MMSE is not a diagnostic tool for ILD. Its potential value in the ILD clinic may instead lie as a rapid screening instrument to identify patients who could benefit from more comprehensive neuropsychological evaluation and integrated care, addressing both pulmonary and cognitive health. Future cost-effectiveness analyses are needed to define its optimal role in clinical pathways.

Our study has several important limitations. First, its cross-sectional nature precludes causal inference. Second, the MMSE, while a validated global cognitive screen, is insensitive to subtle or domain-specific deficits; future studies should employ detailed neuropsychological batteries. Third, despite matching and adjustment, residual confounding by factors such as medication effects, subclinical cerebrovascular disease, or socioeconomic status cannot be excluded. Furthermore, despite statistical adjustment, residual confounding by unmeasured or imperfectly measured vascular risk factors (e.g., the baseline imbalance in hypertension) cannot be entirely ruled out. Fourth, the transcriptomic analysis was performed on a separate cohort without cognitive phenotyping; thus, any link to cognition remains speculative and requires direct validation. Finally, the sample size, though reasonable for an initial study, limits subgroup analyses and generalizability.

Future longitudinal studies with serial cognitive, pulmonary, and multimodal biomarker assessments (e.g., neuroimaging, systemic inflammation, microbiome profiling) are essential to determine the trajectory, reversibility, and mechanisms of cognitive impairment in ILD. Investigating whether pulmonary rehabilitation, oxygen therapy, or anti-inflammatory treatments can ameliorate cognitive impairment is a critical next step. In conclusion, our study suggests that cognitive screening may be a valuable component of holistic ILD management. The potential interplay between pulmonary pathology, systemic inflammation, the microbiome, and brain function opens new avenues for research aimed at improving the comprehensive care and quality of life for patients with ILD.

## Data Availability

The raw data supporting the conclusions of this article will be made available by the authors, without undue reservation.
